# Psychometric properties and convergent and predictive validity of an executive function test battery for two-year-olds

**DOI:** 10.3389/fpsyg.2014.00733

**Published:** 2014-07-22

**Authors:** Hanna Mulder, Huub Hoofs, Josje Verhagen, Ineke van der Veen, Paul P. M. Leseman

**Affiliations:** ^1^Department of Special Education, Faculty of Social Sciences, Utrecht UniversityUtrecht, Netherlands; ^2^Department of Epidemiology, Faculty of Health Medicine and Life Sciences, CAPHRI School for Public Health and Primary Care, Maastricht UniversityMaastricht, Netherlands; ^3^Kohnstamm Institute, University of AmsterdamAmsterdam, Netherlands

**Keywords:** executive function, toddlers, psychometrics, validity, delay of gratification, working memory, selective attention

## Abstract

Executive function (EF) is an important predictor of numerous developmental outcomes, such as academic achievement and behavioral adjustment. Although a plethora of measurement instruments exists to assess executive function in children, only few of these are suitable for toddlers, and even fewer have undergone psychometric evaluation. The present study evaluates the psychometric properties and validity of an assessment battery for measuring EF in two-year-olds. A sample of 2437 children were administered the assessment battery at a mean age of 2;4 years (*SD* = 0;3 years) in a large-scale field study. Measures of both hot EF (snack and gift delay tasks) and cool EF (six boxes, memory for location, and visual search task) were included. Confirmatory Factor Analyses showed that a two-factor hot and cool EF model fitted the data better than a one-factor model. Measurement invariance was supported across groups differing in age, gender, socioeconomic status (SES), home language, and test setting. Criterion and convergent validity were evaluated by examining relationships between EF and age, gender, SES, home language, and parent and teacher reports of children's attention and inhibitory control. Predictive validity of the test battery was investigated by regressing children's pre-academic skills and behavioral problems at age three on the latent hot and cool EF factors at age 2 years. The test battery showed satisfactory psychometric quality and criterion, convergent, and predictive validity. Whereas cool EF predicted both pre-academic skills and behavior problems 1 year later, hot EF predicted behavior problems only. These results show that EF can be assessed with psychometrically sound instruments in children as young as 2 years, and that EF tasks can be reliably applied in large scale field research. The current instruments offer new opportunities for investigating EF in early childhood, and for evaluating interventions targeted at improving EF from a young age.

## Introduction

Executive function (EF) involves a wide array of cognitive processes needed for goal-directed behavior and self-regulation. In children and adults, EF has been shown to involve at least three main components: (i) working memory, defined as the ability to hold information in memory while performing mental operations on this information; (ii) inhibitory control, defined as the ability to suppress automatized and predominant responses; and (iii) shifting, or the ability to change cognitive set in order to switch between different tasks (Miyake et al., 2000; Davidson et al., 2006; Garon et al., 2008). There is growing evidence that EF is a strong predictor of various aspects of child development, such as academic skills. Specifically, studies have found that EF ability at preschool age predicts later academic achievement (Blair and Razza, 2007; Clark et al., 2010). Moreover, development of EF over the preschool years, or growth in EF, is related to growth in academic skills such as math, vocabulary and emergent literacy (Mcclelland et al., 2007; Raver et al., 2011; Van der Ven et al., 2012). Finally, EF is important for more general learning-related skills, such as work attitude (Blair et al., 2005; Ponitz et al., 2009), and socioemotional skills (Denham et al., 2012). Given the importance of EF at a young age for later academic and behavioral functioning, there is a clear need for valid and psychometrically sound instruments to assess EF in early childhood. To date, however, few EF tasks are available for use with children younger than 3 years of age, and the instruments that are available most often have not been evaluated psychometrically. Such a psychometric evaluation is crucial as “the results will only be as good as the test,” which entails that only valid and reliable assessment tools will contribute to our understanding of young children's EF and thereby help to prevent academic failure from a young age (Blair and Diamond, 2008).

Although many studies have investigated EF in preschoolers aged between 3 and 5 years in the past years (Wiebe et al., 2008, 2011; Willoughby et al., 2010) not much is known about EF development in toddlers (cf. Garon et al., 2008). In particular, two-year-olds are a neglected group in research on EF development. Rose et al. (2009) noted that there is a gap in our knowledge about cognitive development in toddlerhood, and others even have described the period between 2 and 3 years of life as the “dark ages” of cognitive development (Meltzoff et al., 1999). One of the reasons for this gap in the literature is undoubtedly the relative difficulty of testing toddlers (see also Hughes and Ensor, 2005). Children this young generally have short attention spans, limited motor skills, and they do not yet dispose of complex language skills. As such, EF measures designed for preschoolers tend to be too challenging for toddlers. Thus, in order to assess rather complex processes such as controlling a dominant response or updating information in memory, tasks have to be developed that measure these abilities while not burdening children's motor, attentional and linguistic skills too much.

For two-year-old children, a few studies have looked at (the development of) EF and/or the relationships with other developmental domains such as theory of mind (Carlson et al., 2004; Hughes and Ensor, 2005, 2007; Miller and Marcovitch, 2011; Fitzpatrick and Pagani, 2012). With some exceptions (Hughes and Ensor, 2005, 2007; Fitzpatrick and Pagani, 2012), most studies have included relatively small samples of children that were tested in highly controlled laboratory settings. Consequently, there often is a high overrepresentation of children from motivated, high socioeconomic status (SES) parents willing to participate in a study, which seriously limits most studies' external validity (see also Willoughby et al., 2010). Also, a close psychometric scrutiny of the EF assessments used in these studies is generally absent.

An exception to this is a study by Carlson (2005), who addressed the psychometric properties of EF assessment tools in two- to six-year-olds, including a sample of 118 two-year-olds. This study showed relatively strong discriminatory power for most tasks for toddlers, enabling a proper differentiation between children of varying EF ability. The sample consisted of children from predominantly middle-class Caucasian families, however. Likewise, Garon et al. (2013) evaluated a battery of tasks assessing working memory, inhibition, and shifting for children aged 18–67 months. This study showed that the EF battery was sensitive to developmental improvements across this age span, and internal consistency of each of the measures was adequate to good. Again, however, the sample contained mostly middle-class families, leaving unanswered the question as to how appropriate such measures are for children from different socio-economic and ethnic backgrounds. Thus, although a few previous studies have assessed the psychometric properties of EF measures in samples with toddlers, these studies included mostly relatively high SES families, leaving unclear how appropriate such tasks are for children from less advantaged backgrounds.

The current study adds to the available literature on the psychometric quality of EF tasks for young children by investigating the psychometric properties of a battery of EF measures in a large sample of two-year-olds from diverse socio-economic and ethnic backgrounds. The EF battery in our study was designed for the purposes of evaluating effects of preschool education and care in the Netherlands on later socio-emotional, cognitive and academic skills (cf. Pre-COOL, see below). Therefore, we aimed to include a set of measures which would predict child developmental outcomes across multiple domains. Our decisions were informed by the literature about the development and factor structure of EF in young children as well as the predictive value of EF tasks for future academic and socio-emotional skills. The factor structure of EF has typically been investigated used Confirmatory Factor Analysis (CFA), a statistical approach which allows for modeling of shared variance amongst constructs. Through using CFA, conclusions can be drawn about the way different tasks cluster together, providing information about the underlying, latent factors which drive task performance rather than specific tasks. In the next sections, we describe two different lines of research on EF in child development, their main findings on the organizational structure of EF in young children, and the predictive validity of the EF construct(s) for developmental outcomes that guided our decisions when designing our task battery.

Miyake et al. (2000) have shown that the structure of EF comprises separate but interrelated inhibition, shifting, and working memory factors in adults. In a recent revision of their theory, however, Miyake and Friedman (2012) showed that EF in adults is best represented by a common EF factor and separate updating-specific and shifting-specific factors. So, their previous inhibition factor now fully overlaps with the common EF factor in this account. Although differentiation of EF into the three components of inhibition, shifting, and working memory in children has been confirmed by Lehto et al. (2003), a number of other studies support a two-factor over a three-factor model in childhood (Van der Ven et al., 2013; Usai et al., 2014). Van der Ven et al. (2013) argued that measurement selection, which varies widely across the EF literature, may be at the core of the variation in findings between studies. A similar pattern of findings occurs in the preschool EF literature. While some previous studies have found a single latent EF construct in preschoolers (Wiebe et al., 2008, 2011), others have observed more differentiated EF skills already at this young age (Garon et al., 2013). Miller et al. (2012) studied the factor structure of EF in three- to five-year-old children using working memory, inhibitory control, and shifting measures. In their first set of analyses, they replicated the finding by Wiebe et al. (2008) that EF comprises a single latent factor at this young age. However, in a second set of analyses, they selected different response indicators for some of their measures and found that a two-factor model with separate but related working memory and inhibition factors fitted their data better than a single or three-factor model. Miller et al. (2012) concluded that measurement and response indicator selection is crucial and may explain different findings across studies, in line with the claims made by Van der Ven et al. (2013). More clear-cut evidence regarding the role of age in the development of the structure of EF across development comes from studies which have administered the same EF battery to children of different ages and investigated measurement invariance across age. For example, Wiebe et al. (2008) found that a unitary EF model fitted their data best in a study of 2.3- to 6-year old children including a comprehensive battery of inhibitory control and working memory measures. They found that their measurement model was invariant across age, indicating that a unitary factor fitted the data well for both younger and older preschoolers. Moreover, Shing et al. (2010) studied a battery of inhibitory control and working memory tasks in children aged 4–14.5 years old. They observed increasing fractionation of EF with age; a single latent factor was observed for their two youngest age groups, while separate working memory and inhibitory control factors were observed in their oldest age group. Thus, although differences in tasks and/or response selection may to a large extent explain differences in findings between studies regarding the fractionation of EF in preschoolers and older children, there is some evidence that EF is a unitary factor in preschool children and only becomes more fractionated when children grow older.

In a separate line of research, a distinction has been made between executive processing of neutral cognitive and affective stimuli. The former typically involve measures of inhibition, shifting, and working memory as described above. The latter is most often limited to assessments of inhibitory control in the face of an affective stimulus, and is usually assessed with delay of gratification tasks, which require the child to suppress touching an attractive object or sweet (Kochanska et al., 2000). Confirmatory factor analyses in studies of young children have shown that the executive processing of cognitive and affective stimuli are typically represented by separate latent factors, labeled “cool” and “hot” EF, respectively (Brock et al., 2009; Willoughby et al., 2011; Bassett et al., 2012). Again, however, there is a discrepancy between studies. While most studies have found that a two-factor model with separate hot and cool factors fitted the data best (Brock et al., 2009; Willoughby et al., 2011; Bassett et al., 2012), others have found that a two-factor model does not fit the data better than a single EF factor model in preschoolers (Allan and Lonigan, 2011). However, investigations of the predictive validity of cool and hot EF as separate factors lend support to their differentiation. In a study by Willoughby et al. (2011), cool EF was predictive of academic performance, while hot EF was predictive of behavioral adjustment in preschoolers. Similarly, Kim et al. (2013) showed that latent cool and hot EF factors differentially predicted academic skills and behavior problems: Cool EF predicted academic performance, while hot EF predicted behavior problems. In contrast to the studies by Willoughby et al. (2011) and Kim et al. (2013), Brock et al. (2009) found that cool EF predicted learning-related behaviors, classroom engagement, and math skills in kindergarteners, while hot EF predicted none of these outcomes when analyzed concurrently with cool EF. Thus, although the differentiation between hot and cool EF is not always confirmed and the theoretical debate about the meaning of this distinction is still ongoing (Welsh and Peterson, 2014), there are clear indications that hot and cool EF measures differentially predict developmental outcomes. An important question that remains is whether hot and cool EF can be distinguished already before preschool age and whether they are differentially predictive of developmental outcomes at this young age, given that all previous studies are on older children.

Since hot and cool EF measures may be differentially predictive of academic and behavioral outcomes, we included measures of hot as well as cool EF in our task battery for toddlers. For each domain, we selected multiple measures, to be able to use CFA and work with latent factors. As argued above, the main advantage of this approach is that task-specific measurement error can be partialled out from the latent constructs under investigation, which is especially beneficial in studies on young children, where measurement error tends to be large. For example, Willoughby et al. (2010) have shown that the association between EF measures and parent, teacher, and research assistant ratings of hyperactivity in three-year olds were weak to moderate for separate test measures, but when EF was modeled as a latent factor, the association with informant ratings of hyperactivity became much stronger. Willoughby et al. conclude that, as the separate measures are confounded with measurement error and task-specific demands (e.g., motor or verbal skills) and the latent factor represents only shared variance across measures, the latent measures provides a more reliable estimate of EF ability. Further evidence comes from test-retest analyses of an EF battery for four-year-olds, showing that test-retest reliability was much higher for a latent EF ability construct than for each separate measure alone (Willoughby and Blair, 2011). Based on these findings, we decided to include multiple measures of the hot and cool constructs in our EF battery. In the cool EF domain, we included two tasks assessing working memory and one task measuring selective attention. We initially also included an inhibitory control task (an adaptation of the Shapes task, Kochanska et al., 2000), but this task proved to be too difficult for the younger children in our sample and was dropped from the battery. In the hot EF domain, we included two delay of gratification tasks, a snack and gift delay (Kochanska et al., 2000).

### Aims of the current study

The aims of our study were twofold: (1) investigate the psychometric properties of our EF task battery for toddlers, and (2) study criterion, convergent, and predictive validity of the test battery. To evaluate the psychometric properties of the test battery, the following steps were taken. First, we applied CFA to evaluate a two-factor hot and cool measurement model and compare this model to a one-factor model. Based on the previous studies described above, we expected to find support for the two-factor over the one-factor model. Second, as children in our sample were either tested at their daycare center or at home, and comprised a mixed group in terms of their language background and SES, we studied whether our measurement model was invariant across a number of groups: SES (low/middle vs. high SES), age (<2.5 years vs. >2.5 years of age), assessment setting (home vs. day care center), home language (monolingual Dutch vs. non-monolingual Dutch), and gender. If measurement model invariance is supported across groups, this implies that measures relate to the latent constructs in the same way across different groups, allowing for a fair comparison between different subgroups of children.

Criterion validity was studied by examining relationships between children's latent EF abilities and gender, SES, home language, age, and assessment setting. Previous studies investigating gender differences in EF have yielded mixed results, with some studies showing that girls outperformed boys (Kochanska and Knaack, 2003; Wiebe et al., 2008), and others showing no gender differences (Wiebe et al., 2011). Two recent studies have investigated gender differences in EF in children across different cultural contexts, using the Head-Toes-Knees-Shoulders task (Ponitz et al., 2009). Gender differences were observed in the United States and Iceland, but not in Taiwan, China, South Korea, Germany, and France, suggesting that cultural differences in socialization practices might play a role in the emergence of gender differences in EF (Wanless et al., 2013; Gestsdottir et al., 2014). Not much is known about gender differences in EF in children in the Netherlands, although Huizinga and Smidts (2011) found that Dutch five to 18-year-old girls received higher ratings on EF by their parents than boys. Therefore, we expected that, if a gender difference was observed, girls would perform better than boys. Furthermore, children from lower SES families were expected to obtain lower scores than children from high SES families (Hughes and Ensor, 2005; Noble et al., 2005, 2007). As for home language, no clear prediction could be formulated. Previous studies have shown that bilingual children may show enhanced EF as compared to their monolingual peers, already at preschool age, but in young children, this EF advantage seems to be restricted to native bilingual children who are exposed to two languages at home from birth (Blom et al., in press; Carlson and Meltzoff, 2008; Poulin-Dubois et al., 2011). In our sample, a large number of children were predominantly exposed to another language than Dutch at home. These children may score lower on the EF tasks which were administered in Dutch, due to their poorer knowledge of the Dutch language. In addition, associated with a different home language, different cultural customs regarding early play and cognitive stimulation can be at stake that can influence EF scores. As for age, previous studies have shown significant growth in EF skills during the third year of life (Garon et al., 2013). Therefore, we expected a strong effect of age on EF ability.

Convergent validity of the test battery was assessed through studying the association between children's EF ability and parent and teacher reports of children's attention and inhibitory control. Parent- and teacher-rated attentional focusing and inhibitory control scores were expected to be positively related to children's EF scores, as these two temperament dimensions are conceptually related to EF (Rothbart et al., 2003; Blair and Razza, 2007). Finally, predictive validity was assessed by regressing children's pre-academic skills and behavior problems at preschool age on children's EF scores at toddler age. Whereas hot EF was expected to predict behavior problems, cool EF was expected to predict pre-academic skills (Willoughby et al., 2011; Kim et al., 2013).

## Methods

### Participants

Children participating in this study were involved in the longitudinal national cohort study Pre-COOL on the effectiveness of preschool care and educational provisions in the Netherlands, commissioned by the Dutch Ministry of Education, Culture and Sciences (Veen et al., 2012). In Pre-COOL, children are being assessed longitudinally from age 2–5 years. At the first wave of assessment, children were aged 2 years (*M* = 2;4 years, *SD* = 0;3 years, range = 1;8–3;1 years). Although the age range was wide, 70% of children were aged between 2;0–2;6 years, 28% were aged between 2;6 and 3;0 years, only 22 children were below 2;0 years (<0.01%), and six children were older than 3;0 years (<0.01%). At the second measurement wave, children were aged 3;6 years on average (*SD* = 0;2; range 2;11–4;5 years). The average time interval between assessments was 1;2 years (*SD* = 0;4; range 0;6 to 2;2 years). Gender was equally distributed (49% girls). As for SES, 41.5% of the children were from low/middle SES families and 58.5% came from high SES families. Most children were from monolingual Dutch homes; 28% of the children came non-monolingual Dutch families. The sample consisted of two sub-samples: a center-based sample which included children participating in center-based education and care and recruited through their center, and a home-based sample which included children recruited through the municipal registration records (and as such includes both children attending day care and children not attending day care). The sample was geographically well-spread across rural, semi-urban, and urban areas in all parts of the Netherlands. Approval for the study was obtained from the Ethical Advisory Committee of the Faculty of Social and Behavioral Sciences of Utrecht University^1^.

#### Center-based sample

The Pre-COOL study is linked to the national cohort study COOL. The latter is aimed at following students' educational careers in Dutch primary and secondary education from age 5 to 18 years. Children in the Pre-COOL sample will enroll in the COOL study, so they can be followed from toddler age through to late adolescence. To increase the likelihood of Pre-COOL participants entering primary schools involved in COOL, recruitment of the center-based sample proceeded in a number of steps. First, primary schools participating in COOL were selected. 300 primary schools, randomly drawn from the COOL cohort, were approached. 139 schools agreed to participate. Next, COOL primary schools were asked to identify the preschool day care and education centers that were attended by most of their new students. In addition, municipal records and the internet were used to identify additional preschool care and education centers in the same neighborhoods as the COOL schools. Over 500 centers across the Netherlands were invited to participate in Pre-COOL, of which 289 centers agreed to take part. Finally, children born between April 1 and November 1, 2008, were identified in these centers. Parents of eligible children were personally informed by their child's teacher about the Pre-COOL study and were given a letter containing information about the study, explicitly giving them the opportunity to withdraw their child from participation by notifying the teacher. In total, 1819 children enrolled in the center-based sample.

#### Home-based sample

A sample of 6000 families with a child born between April 1 and November 1, 2008, living in neighborhoods close to the participating COOL schools was drawn from the municipal population registers. Parents received a letter in which they were invited to take part in the study with a pre-paid answering card. Additionally, families with an immigration background living in Pre-COOL neighborhoods in the urban agglomerations of Amsterdam, Rotterdam and The Hague were contacted personally during home visits in order to increase participation from these groups. In total, 1139 parents responded to the study invitation. Of those, 1008 agreed to participate in the study.

### Procedures

Children participating in the study were assessed at home (home-based sample) or at their center (center-based sample) at both waves. Testing took place in a quiet room. The tests in this study were part of a more comprehensive test battery which took on average 45 min to administer. Tests were given in a fixed order. At the first wave, two computerized language tasks, the visual search task, two further computerized language tasks, the snack delay, memory for location, six boxes, and gift delay task were given. At the second wave, a computerized language task, the vocabulary task, visual search task, two further computerized language tasks, and a computerized EF task, gift delay, emergent math, six boxes, and a second delay task were given. Research assistants (RA's) allowed children to have short breaks when necessary. Parents and teachers were asked to fill out a questionnaire with items addressing, among others, demographic variables and children's temperament and behavior.

To secure standardized assessment, RA's went through an intensive training phase before they were allowed to start data collection in each study wave. First, they attended a full day test administration course. Second, they received a very detailed standardized test protocol with step-by-step descriptions of the procedures for each measure. Third, they submitted a video recording of a practice session with a two-year-old to the study center, together with their scoring forms. The test administration procedures and scoring forms were carefully reviewed by the first and third author, and each RA was sent a detailed feedback report. This report was discussed by telephone. If the RA followed the standardized protocol, they were allowed to start data collection. If major administration or coding errors were observed, the RA was required to submit a second video for feedback purposes. The first and third author discussed any difficult cases until agreement was reached, and read each other's feedback reports before sending them to RA's, to ensure that no divergence in their evaluations occurred throughout the process.

### Measures

At the first wave, children completed the EF tasks, parents rated children's inhibitory control and attentional focusing, and teachers rated children's inhibitory control and work attitude in the classroom. At the second wave, children's emergent math skills and vocabulary were assessed, and parents and teachers rated children's externalizing behavior problems. Each of the measures is described below in turn. It should be noted that teacher ratings were only available for the center-based sample.

#### Wave one measures

***Attention (visual search)***. To measure selective attention, a computerized visual search task was developed for the purposes of the present study, based on the work by Gerhardstein and Rovee-Collier (2002), and Scerif et al. (2004). In this task, children were shown a structured display of 48 animals on a 6 × 8 grid on the laptop screen using E-Prime 2.0 (Schneider et al., 2002). Stimuli were images of elephants, bears, and donkeys, which were the same in color and size. Children were instructed to locate as many targets (elephants) as possible while ignoring the distractors (bears and donkeys). As such, children had to try to focus their attention only on the targets while suppressing interfering visual stimuli. To minimize memory demands, the targets that the child had located were crossed off with a line by the assessor. Following three practice trials, children were given three test items which lasted 40 s each. Each test item contained eight targets. Throughout the test items, children were encouraged to search as fast as possible and were continuously given feedback according to protocol (i.e., when the child pointed to a target: “Well done! Can you find another elephant?” or when the child pointed to a distractor: “No, where is an elephant?” or when the child pointed to the same elephant twice: “No, where is another elephant?”). Feedback rules were developed following careful piloting. Corrective feedback was used to ensure memory demands of this task were minimal. Accuracy for each test item was scored and averaged across items (i.e., the number of targets located correctly within the time limit, range 0–8). When children achieved a total score of “0”, indicating that they did not find any targets on the three test items, their score was set to “missing,” as we cannot be completely certain that they understood the task rules properly.

***Visuospatial working memory task (six boxes)***. The six boxes task (Diamond et al., 1997) was used to measure visuospatial working memory capacity. To familiarize children with the task, a practice trial was given in which the child was shown how two wooden toys were hidden in two identical white boxes with blue lids. The child was then instructed to retrieve the toys one by one. The RA distracted the child for 1 s in between the two search attempts. If the child failed the practice trial (i.e., the child didn't find both toys in two search attempts), this procedure was repeated. After the practice trials, the test trials were given.

For the six test trials, six different wooden toys were hidden in six identical white boxes with blue lids while the child watched. The boxes were placed in two slightly asymmetrical rows of three boxes, rather than two perfectly aligned rows, to discourage the use of a simple strategy of opening the boxes row by row. Children were given six search attempts to find all toys. They were actively distracted by the RA for 6 s in between search attempts, as pilot work had shown that a 6 s delay gave the most optimal distribution of scores for this age range. After the child had taken a toy out of a box, the RA showed them clearly that that box was empty before closing the lid again (“Look, this one is empty now!”). If children moved a box without opening it (for example, by shaking it lightly to hear if it contained a toy), the RA opened the box and this box was scored as the child's choice for that search attempt. On both the practice and test trials, children were given positive feedback when they opened a box containing a toy. However, when they opened a box that was already empty, they were told “Oh no, that one is empty” to encourage them to search in a different box at the next search attempt. Thus, in this task, children had to try to remember which boxes they had already emptied and which boxes still contained a toy and retain this information over the delay time. Accuracy across test trials (i.e., the number of toys obtained correctly) was scored for each child.

***Visuospatial short-term memory span task (memory for location)***. This task assesses visuospatial memory span and was based on work by Pelphrey et al. (2004) and Vicari et al. (2004). The procedure of this task was similar to that of the six boxes task: Children were shown how a different set of small wooden figures was hidden in six identical white boxes which were placed in two symmetrical rows of three boxes each. However, in contrast to the six boxes task, the number of figures hidden varied across test items (range 1–4). After hiding the figures, the RA distracted the child for 1 s, and the child was then asked to find all the figures for that item. An item was scored as correct if the child retrieved all hidden figures in the minimum number of search attempts.

For this task, an adaptive testing procedure was used in which task difficulty level increased after each successful item. Difficulty level was defined as the number of hidden figures and ranged from one to four. This difficulty level was based on previous work showing that 24-month old toddlers were able to hold between two and three items in memory (Rose et al., 2009), and our own pilot work with children between age 2 and 3 years.

On the first test item, one figure was hidden. If the child passed this item, difficulty level was increased, and two figures were hidden on the next item. However, if the child failed the first item, an additional item with one figure was given. Children received up to two trials for each difficulty level, with the exception of the first level for which children received up to three trials to familiarize them with the procedure. If children failed all items at a given difficulty level, task administration was discontinued. Throughout the task, children were given feedback in a similar fashion as during the six boxes task (“Well done!” when they found a toy and “Oh no, that one is empty” when opening a box which did not contain a toy). The number of locations that a child could retain in memory simultaneously was measured in this task. Scores were calculated as the highest level (i.e., span) performed correctly for each child (range 0–4).

***Delay of gratification (snack delay)***. The snack delay task was a simplified version of the Kochanska et al. (2000) snack delay task. In this task, an open box of raisins was placed in front of the child on the table at a distance of 25 cm. The child was then instructed to try not to touch the box of raisins until the RA had finished another task. The RA then moved away out of sight of the child and observed the child's behavior for 1 min. After the delay time, the child was always given positive feedback and they were given the box of raisins (if they had not already taken the box themselves). Three different behaviors were coded by the RA during the delay time: (1) touching the box or raisins, (2) picking up the box or raisins, and (3) eating the raisins. The occurrence of each behavior was coded as present (0) or absent (1) during the delay, so that a higher score indicated better task performance. The sum across these behavioral codes was scored (range 0–3). Children who obtained a total score of 1 or 2 were collapsed into one group due to a low number of children obtaining these scores (i.e., most children either ate the raisins or refrained from touching them). The total score then ranged from 0–2.

***Delay of gratification (gift delay)***. The gift delay task was an adaptation of the Kochanska et al. (2000) gift delay task. This task was similar to the snack delay task, except that the box of raisins was replaced by an attractively wrapped gift with a bow. The child was instructed to try not to touch the gift during a delay of 1 min. The occurrence of three different behaviors was coded by the RA during the delay time: (1) touching the gift or bow, (2) tearing the wrapping paper, and (3) unpacking the gift completely (i.e., by taking the gift, a small rubber duck, out of the wrapping paper). However, the third category, unwrapping the gift completely, turned out to be too demanding for the motor skills of children this young, and was omitted from the analyses. The occurrence of each of the remaining two behaviors was coded as present (0) or absent (1) during the delay time of 1 min, so that a higher score indicated better task performance. The total score for this task was the sum across these behavioral codes (range 0–2).

In a separate study, video observations of the snack and gift delay tasks were coded to determine the reliability of the live codes in a sample of Dutch two- and three-year-olds. Kappa's were as follows for the snack delay task (*N* = 59): 0.96 for touching behavior and picking up the box of raisins combined, and 0.90 for eating the raisins. Agreement between video and live codes was 98.3 and 96.6%, respectively (chance level of agreement: 50%). For the gift delay task, the following Kappa's were observed (*N* = 53): 0.89 for touching behavior, and 0.74 for tearing the wrapping paper. Agreement between video and live codes was 96.2 and 94.3%, respectively (chance level of agreement: 50%).

***Parent and teacher ratings of inhibitory control and parent ratings of attentional focusing (Early Childhood Behavior Questionnaire)***. The parent and teacher rated constructs inhibitory control and attentional focusing were assessed using a shortened version of the Dutch version of the Early Childhood Behavior Questionnaire (ECBQ, Putnam et al., 2006). This questionnaire was filled out by children's parents (six items for inhibitory control, four items for attentional focusing) and one of their teachers (three items for inhibitory control). As participating children in the center sample were often in the same group, many teachers had to fill out the questionnaire for more than one child in their group. Thus, very few items were selected for use with teachers to keep the questionnaire as short as possible. Items were selected based on pilot work with 56 parents and 44 teachers of two- to three-year-olds. Although the ECBQ is originally designed for use with parents (Putnam et al., 2006), we made minimal adaptations to questionnaire items for use with daycare teachers (i.e., “your child” in the parent questionnaire was replaced by “this child” in the teacher questionnaire'). For each item, respondents were asked to indicate the frequency with which a certain behavior (e.g., “ignoring a warning”) occurred on a seven-point Likert scale (from “never” to “always”). Example items are: “When told no, how often did your/this child ignore your warning?” (inhibitory control) and “When engaged in an activity requiring attention, such as building with blocks, how often did your child stay involved for 10 min or more?”(attentional focusing). Cronbach's alpha's were 0.78 and 0.84 for parent and teacher rated inhibitory control, respectively, and 0.78 for parent rated attentional focusing.

***Teacher ratings of children's attention***. Teachers of children in the center cohort also reported on children's attention during play and work using a four-item scale based on a short questionnaire designed for the COOL study (Driessen et al., 2009) and the SCHOBL-R (Bleichrodt et al., 1993). This tool has been found to be appropriate for collecting data on children's behavior in center-based care and education settings. Items concerning classroom behaviors (e.g., “works carefully,” “is attentive”) were rated on a five-point Likert scale (from “definitely untrue” to “definitely true”). Cronbach's alpha was 0.80.

#### Wave two measures

***Parent and teacher ratings of children's externalizing behavior problems***. To assess children's externalizing problem behavior, caregivers were asked to rate five items of the Problem Scale of the Brief Infant-Toddler Social and Emotional Assessment (BITSEA) (Briggs-Gowan and Carter, 2001). The following aspects of externalizing problem behavior are included in the BITSEA: activity/impulsivity, aggression/defiance, and peer aggression. The selection of items from the original Problem Scale was based on pilot data. Criteria were: inclusion of all three topics, discriminatory power, good internal consistency, and suitability of items for both parents and caregivers. Example items are: “Is very loud” (activity/impulsivity), “Purposely tries to hurt you (or other parent)” (aggression/defiance), and “Hits, shoves, kicks, or bites children (not including brother/sister)” (peer aggression). Cronbach's alpha was 0.85 for teachers and 0.86 for parents.

***Children's emerging math skills***. Children's emergent math skills were assessed with a short version of the Math Test for Toddlers developed by the Dutch National Institute for Educational Measurement (CITO) (Op den Kamp and Keuning, 2011). About two thirds of the total number of test items (15) were selected by CITO, based on suitability of difficulty, discriminatory power and adequacy of reliability (of 0.70). To gain a more even distribution of items across topics/aspects, one item was added. The final selection of 16 items covered three aspects: number sense, measurement, and geometry. Using item response theory (IRT) modeling, a skill score was calculated by CITO based on the responses to the 16 items.

***Children's vocabulary***. Receptive vocabulary was assessed with the Dutch version of the Peabody Picture Vocabulary Test (PPVT-III-NL, Dunn and Dunn, 2005). In this test, children were asked to select one out of four picture drawings after an orally presented word. Whereas this task is usually performed as a paper-and-pencil task, stimuli presentation in the current study was controlled by the experimental software E-Prime 2.0 (Schneider et al., 2002), and administered through a laptop computer to facilitate administration and scoring. The shortened version used in our study contained eight items per test set, instead of the usual twelve items, due to testing time constraints. Sets 3, 4, and 5 were presented. As each set contained eight items, there were 24 items in total. Pilot research with 97 three-year-olds established that the items that were removed did not differentiate well among children, as they were either very easy or very difficult (i.e., mean scores on these items were either below 30% or above 70% correct). Scores were calculated as the percentage of correct responses for each child.

#### Background variables

***Socioeconomic status***. Parental education was used as an indicator of SES. In two-parent households, parental education of the parent with the highest education was taken as a proxy for family SES. Intermediate vocational education or lower were coded as low to middle SES, while a higher vocational college or University education were coded as high SES. SES information was collected through parent questionnaire at the first study wave; if parent reports were missing at this wave, parents were asked to report SES in subsequent study waves. SES was available for 1843 children (65%).

***Home language***. Parents reported on children's home language in the parent questionnaire. For the purposes of the present study, we coded whether Dutch was the only language children were exposed to at home or whether they were (also) exposed to (an)other language(s). As the parent questionnaire was missing for a large number of children (see sample description section below), we asked assessors to record children's home language as well at both waves. Assessors were instructed to enquire after children's home language with the parents in the home-based sample and with teachers in the center-based sample. When parent questionnaire data were not available, the wave one assessor's report of home language was imputed. In cases where wave one assessor reports were also unavailable, wave two reports were used. When wave one and wave two assessor reports provided conflicting information, the home language variable was set to missing. Home language information was available for 2463 children (87%).

### Analytic strategy

First, we investigated model fit of a one-factor and two-factor (hot vs. cool) EF model using CFA. The fit of the CFA models was assessed with the comparative fit index (CFI) and the root mean square error of approximation (RMSEA) (Kline, 2011). CFI values greater than 0.90 and RMSEA values of less than 0.08 were considered as acceptable fit (Hu and Bentler, 1999). CFI values greater than 0.95 and RMSEA values of less than 0.05 were considered as good fit (Schreiber et al., 2006). As χ^2^ is not appropriate for investigating model fit when the sample size is very large, we only report χ^2^ for the sake of clarity. The best fitting model was selected for further analyses.

Second, multi-group CFA models were used to evaluate measurement invariance of the EF model, with gender (boys vs. girls), age (below 2.5 years vs. above 2.5 years), home language (monolingual Dutch vs. other), SES (low/middle vs. high), and test setting (home vs. daycare center) as grouping variables. Measurement invariance was investigated by testing the equivalence of factor loadings and thresholds across groups (Millsap, 2011; Muthén and Muthén, 2013). Four nested models were tested successively for each grouping factor. The first model (configural invariance model) had no constraints regarding any parameter across groups. This model was used to evaluate whether the model held for both groups. In the second model (metric invariance model), factor loadings were constrained to be equal for both groups. For identification purposes, the mean of the reference (first) group was fixed to zero and scale factors were fixed to one. Furthermore, the first threshold value of an indicator was constrained to be equal across groups. The intercept/thresholds of the indicator that was used to set the metric of the model was also constrained to be equal across groups. In the third model (scalar invariance model), all factor loadings, intercept, and thresholds were constrained to be equal across groups. Other settings were equal for the second and third model. In the fourth model (factor covariance model), we constrained the association between the latent factors in the two-factor hot and cool EF model to be equal between groups (Schmitt and Kuljanin, 2008).

As the sample size was large, classical difference testing using the χ^2^ was not appropriate (Cheung and Rensvold, 2002; Chen, 2007). Therefore, following recommendations by Chen (2007), we evaluated whether measurement invariance was present by considering changes in CFI and RMSEA. Specifically, a CFI change of 0.01 or less and RMSEA of 0.015 or less indicates measurement invariance for any of the tested sorts; a CFI change above 0.01 and/or RMSEA change exceeding 0.015 indicates measurement invariance is not supported.

Third, we assessed criterion, convergent, and predictive validity of the EF latent factor model in a set of separate analyses. Criterion validity was studied by regressing the latent EF factor(s) on age, gender, home language, SES, and test setting. An alternative approach would have been to compare latent mean factors across groups in the multi-group analyses described above. However, age, home language, SES and test setting were significantly associated with each other. Therefore, a multivariate approach was deemed more appropriate than multigroup comparisons to determine criterion validity. Convergent validity was studied by assessing the association between the latent EF factor(s) and a latent inhibitory control factor, using parent and teacher rated inhibitory control as indicators, and a latent attention factor, using parent attentional focusing and teacher work attitude as indicators. As we were interested in the shared variance within each construct and not in the shared variance within reporters, inhibitory control and attention, reported by parents and teachers, were modeled separately. First, for both the inhibitory control and attention model, separate parent and teacher latent factors were constructed. Next, secondary factors representing the shared variance between parent and teacher reports were modeled and correlated with latent EF. To control for age at assessment, age was entered as a covariate for all latent factors in these models.

Finally, predictive validity was studied by regressing children's latent pre-academic skills, using emergent math skills and vocabulary as indicators, and children's externalizing behavior problems, using parent and teacher ratings as indicators, at age 3 years on the latent EF factor(s) at age 2 years. Age at assessment was controlled for, by regressing the latent EF factor(s) on children's age at wave one, and the latent pre-academic and behavior problem factors on age at wave two. As age at the two waves was significantly associated, the correlation between age at the first and second wave was also included. To make full use of the large dataset, the model was run for the sample as a whole, despite the fact that teacher reports were not available for children in the home cohort. To evaluate whether our findings were robust despite the fact that teacher questionnaire data were missing by design in the full sample, we also tested the model in the center cohort alone.

To investigate the missing data pattern, missingness was analyzed as a function of cohort, home language, and gender. Not enough SES information was available to investigate missingness in relation to SES reliably. We coded missingness on parent and teacher questionnaires and child assessments as the presence or absence of at least one parent questionnaire, teacher questionnaire, and child task score across waves, respectively. Data missingness on child tests and parent questionnaires was significantly associated with cohort and home language, but not gender. There were more missings in children from non-monolingual Dutch families and children in the center cohort on these variables. Missingness on teacher questionnaires was not significantly associated with home language or gender. Given the association between some of the background variables and data missingness, cohort, home language, gender, and SES were entered as covariates in addition to age in each of the validity models. The correlations between these background variables were also included. All available data were used in the analyses; for example, if a child had missing data on one of the EF measures, his or her scores on the other measures were still used in the CFA models. By including the covariates in the validity models, missing data were estimated using the covariates rather than by removing cases, thus preventing estimation bias. All analyses were conducted in Mplus 7.11 (Muthén and Muthén, 1998–2012). As proposed by Byrne and Stewart (2006), WLSMV was used as an estimator in all analyses, because categorical items were present.

## Results

### Sample description and task completion

In total, 2827 children were enrolled in the study. Of those, 390 children (14%) did not complete any of the EF tasks, with task completion defined as responding to at least half of the items of a test. Reasons for not completing a test varied from non-compliance, child illness and language difficulties, to external factors which disturbed the testing situation or technical difficulties. The number of children completing each of the tasks is shown in Table 1. Of the 2437 children who completed at least one of the EF tasks, 64% completed all five tasks, 23% completed four tasks, 8% completed three tasks, 2% completed two tasks, and 4% completed one task. At the second wave, vocabulary scores were available for 2088 children and emerging math scores were available for 2063 children (74 and 73% of the full sample, respectively). There were 2604 children (92% of the full sample) for whom at least one task score (EF, emergent math, and/or vocabulary) was available across waves. Parent reports were available for 1471 children at the first wave and 1351 children at the second wave (52 and 48% of the full sample, respectively). There were 1820 children (64%) for whom at least one parent questionnaire was available. Teacher reports were available for 910 children at the first wave and 904 children at the second wave (50% of children in the center sample). There were 1279 children (70% of children in the center sample) for whom at least one teacher questionnaire was available. There were 171 children for whom no data were available on tasks and questionnaires at both measurement waves (6% of the full sample), 129 children for whom task and/or questionnaire data were present at the second, but not at the first wave (5%), 486 children who had task and/or questionnaire data at the first, but not the second wave (18% loss to follow-up), and 2041 children for whom task and/or questionnaire data were present at both waves (72%).

**Table 1:** **Descriptive statistics for executive function measures**.

**Task**	***N***	**% completion^a^**	***M***	***SD***	**Range**	**Skew (*SE*)**	**Kurtosis (*SE*)**	**Floor (%)**	**Ceiling (%)**
**Continuous measures**									
Visual search	2174	77/89	3.5	1.7	0.3–8	−0.1 (0.1)	−0.8 (0.1)	2.8	0.05
Six boxes	2186	77/90	64.6	18.7	0–100	−0.2 (0.1)	−0.1 (0.1)	0.3	7.6
Memory for location^b^	1803	64/74	2.0	0.9	0–4	0.3 (0.1)	−0.4 (0.1)	2.1	5.4
**Categorical measures**	***N***	**Score distribution (%)**							
		**% completion^a^**	**0**	**1**	**2**				
Snack delay	2298	81/94	29.5	21.3	49.3				
Gift delay	2289	81/94	17.7	29.5	52.8				

a Task completion is shown as: percentage of the total sample (N = 2827)/percentage of the sample who completed at least one test (N = 2437).

b The lower number of children completing the memory for location task was due to the fact that this task was reduced in length after data collection had already begun; data of the first group of children that was assessed were not available for the present analysis.

### Descriptive statistics

Descriptive statistics for each of the EF tasks are shown in Table 1. The visual search, six boxes, and memory for location task did not show strong ceiling or floor effects. The categorical delay task measures showed a less optimal distribution, with about half the sample passing each of the tasks (i.e., not touching the snack or gift). At age 3 years, the mean score of the emergent math task was 40.3 (*SD* = 10.6; range = 2.3–72.6) and the mean of the vocabulary task was 63.7 (*SD* = 18.2; range = 0–100).

Table 2 shows the correlations between each of the continuous EF measures. The visual search, six boxes, and memory for location task scores were significantly correlated with each other in the expected direction, although correlations were weak. Each of the measures was also significantly related to age, as expected given the large age range in our sample. When controlling for the effect of age, the correlations between measures were reduced in strength but remained statistically significant. With respect to the categorical EF measures, there was a significant association between the snack and gift delay task scores [χ^2^_(4)_ = 706.2; *p* < 0.001]. Table 3 shows that performance on both the snack and gift delay task was significantly and positively associated to performance on the visual search, six boxes, and memory for location tasks, also after controlling for the shared variance with age.

**Table 2:** **Correlations between continuous executive function measures**.

	**Visual search**	**Six boxes**	**Memory for location**	**Age**
Visual search	-	0.22^***^	0.25^***^	0.36^***^
Six boxes	0.18^***^	-	0.17^***^	0.20^***^
Memory for location	0.19^***^	0.13^***^	-	0.18^***^

*** p < 0.001. Correlations below the diagonal are partial correlations corrected for age.

**Table 3:** **ANOVA with categorical snack and gift delay task scores as independent variables and continuous executive function measures as dependent variables**.

	**Snack delay (*M, SD*)**			***F* (*df*)**	***F* (*df*)^a^**
	**0**	**1**	**2**		
Visual search	3.2 (1.6)	3.4 (1.6)	3.8 (1.6)	26.7 (2, 2053)^***^	10.4 (2, 2050)^***^
Six boxes	60.5 (19.8)	62.7 (19.1)	67.7 (17.4)	32.8 (2, 2127)^***^	21.6 (2, 2079)^***^
Memory for location	1.8 (0.9)	2.0 (0.9)	2.1 (0.9)	20.9 (2, 1746)^***^	11.9 (2, 1732)^***^
	**Gift delay (*M, SD*)**				
	**0**	**1**	**2**		
Visual search	2.8 (1.7)	3.2 (1.7)	3.7 (1.7)	47.7 (2, 2059)^***^	25.9 (2, 2056)^***^
Six boxes	59.5 (19.7)	62.0 (18.6)	67.6 (17.9)	34.9 (2, 2144)^***^	24.3 (2, 2093)^***^
Memory for location	1.7 (0.9)	1.9 (0.9)	2.1 (0.9)	23.5 (2, 1746)^***^	15.5 (2, 1730)^***^

a ANCOVA analysis with age as covariate.

*** p < 0.001.

### Baseline model

Next, we investigated model fit of both a one-factor and two-factor model, with separate cool and hot EF latent factors specified in the latter. In the two-factor model, the visual search, six boxes, and memory for location tasks were indicators of the cool EF factor, while the snack and gift delay tasks were indicators of the hot EF factor. In both models, age at wave one was included as a covariate. The one-factor model showed poor fit [χ^2^ (9, *N* = 2383) = 326.58, *p* < 0.001, RMSEA = 0.122 (0.111–0.133), CFI = 0.838]. However, model fit of the two-factor model was good [χ^2^ (7, *N* = 2383) = 29.62, *p* < 0.001, RMSEA = 0.037 (0.024–0.051), CFI = 0.988]. For hot EF, standardized factor loadings for the snack delay and gift delay task were 0.77 and 0.86 (*p*'s < 0.001), respectively. For cool EF, standardized factor loadings for the visual search, six boxes, and memory for location task were 0.61, 0.42, and 0.41 (*p*'s < 0.001), respectively. Furthermore, age was a significant predictor of both cool and hot EF (β = 0.53, *p* < 0.001; β = 0.28, *p* < 0.001, respectively). Finally, the cool and hot EF factors were significantly associated (β = 0.44; *p* < 0.001). Because of the better model fit of the one- compared to two-factor model, the two-factor model was used for further analysis.

### Measurement invariance

Next, we investigated whether the two-factor hot and cool EF model showed measurement invariance across subgroups of age, gender, home language, SES, and test setting. For each of these grouping variables, a set of nested models was tested and compared to each other, after constraining an increasing number of parameters. Age was controlled for in all models, except in the model where age was the grouping variable. Model fit was good for all models (CFI > 0.95, RMSEA < 0.05; Table 4). Configural, metric, scalar and factor covariance invariance was supported across all subgroups, as the changes in CFI were never larger than 0.01 and the changes in RMSEA never exceeded 0.015. Thus, the two-factor hot and cool EF model fitted the data well in all groups, and factor loadings and intercepts (continuous variables) and thresholds (categorical variables) of the indicators could be constrained to equality between groups differing in age, gender, home language, SES, and test setting. In addition, the association between the hot and cool factors could be constrained to equality between groups. In sum, the two-factor hot and cool EF model showed strong measurement invariance across age, gender, home language, SES, and test setting groups.

**Table 4:** **Analysis of measurement invariance across groups**.

**Model**		**Model fit indices**			**Nested model comparisons**		
		**χ^2^ (*df*)**	**CFI**	**RMSEA**	**Comp**	**ΔCFI**	**ΔRMSEA**
**AGE (*N* = 2383)**							
1a	Configural	16.62^*^ (8)	0.994	0.030			
1b	Metric	13.89 (11)	0.998	0.015	vs. 1a	0.004	0.015
1c	Scalar	29.06^*^ (14)	0.990	0.030	vs. 1b	0.008	0.015
1d	Factor covariance	32.40^**^ (15)	0.989	0.031	vs. 1c	0.001	0.001
**GENDER (*N* = 2425)**							
2a	Configural	31.70^**^ (14)	0.991	0.032			
2b	Metric	27.73^*^ (17)	0.994	0.023	vs. 2a	0.003	0.009
2c	Scalar	30.17 (20)	0.995	0.020	vs. 2b	0.001	0.003
2d	Factor covariance	29.28 (21)	0.996	0.018	vs. 2c	0.001	0.002
**HOME LANGUAGE (*N* = 2350)**							
3a	Configural	31.88^**^ (14)	0.991	0.033			
3b	Metric	41.93^***^ (17)	0.987	0.035	vs. 3a	0.004	0.002
3c	Scalar	47.28^***^ (20)	0.986	0.034	vs. 3b	0.001	0.001
3d	Factor covariance	49.49^***^ (21)	0.985	0.034	vs. 3c	0.001	<0.001
**SES (*N* = 1772)**							
4a	Configural	29.03^*^ (14)	0.990	0.035			
4b	Metric	26.06 (17)	0.994	0.025	vs. 4a	0.004	0.010
4c	Scalar	30.92 (20)	0.992	0.025	vs. 4b	0.002	<0.001
4d	Factor covariance	32.28 (21)	0.992	0.025	vs. 4c	<0.001	<0.001
**TEST SETTING (*N* = 2524)**							
5a	Configural	28.34^*^(14)	0.993	0.028			
5b	Metric	39.53^**^ (17)	0.988	0.032	vs. 5a	0.005	0.004
5c	Scalar	62.14^***^ (20)	0.978	0.041	vs. 5b	0.010	0.009
5d	Factor covariance	72.64^***^ (21)	0.973	0.044	vs. 5c	0.005	0.003

*** *p < 0.001*,

** *p < 0.01*,

* p < 0.05.

### Criterion validity

To investigate criterion validity, latent hot and cool EF factors were regressed on age, gender, SES, home language, and test setting. Model fit was good [χ^2^ (19, *n* = 2827) = 57.23, *p* < 0.001, RMSEA = 0.027 (0.019–0.035), CFI = 0.987]. Age was positively related to both cool and hot EF, so that older children obtained higher scores than younger children (β = 0.61, *p* < 0.001; β = 0.25, *p* < 0.001, respectively). Also, girls obtained higher scores than boys on both cool and hot EF (β = 0.16, *p* < 0.001; β = 0.10, *p* < 0.001, respectively). Although SES was positively related to cool EF, no effect of SES on hot EF was observed (β = 0.23, *p* < 0.001; β = 0.03, *p* = 0.313, respectively). Children from monolingual Dutch families obtained higher cool and hot EF scores than children from families in which another language next to or instead of Dutch was spoken (β = 0.19, *p* < 0.001; β = 0.08, *p* = 0.004, respectively). Furthermore, children who were tested at their daycare center had higher scores on hot EF than children who were tested at home (β = 0.12, *p* < 0.001). No effect of test setting on cool EF was observed (β = −0.009, *p* = 0.783).

### Convergent validity

**Figure 1:**
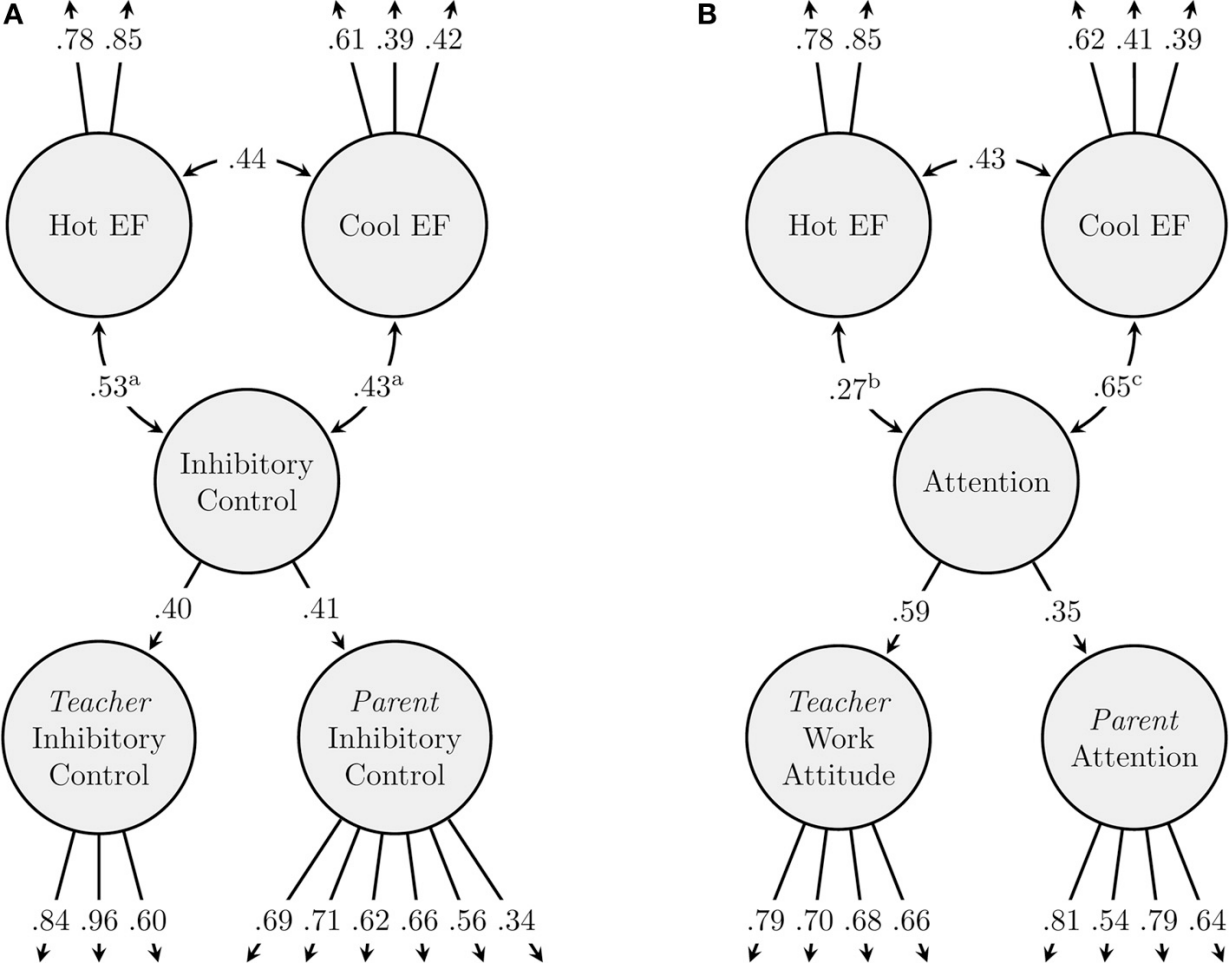
**(A)** Convergent validity model: Association between cool and hot EF and report-based inhibitory control. Age, gender, SES, home language, and cohort were included as covariates in the model. **(B)** Convergent validity model: Association between cool and hot EF and report-based attention. Age, gender, SES, home language, and cohort were included as covariates in the model.

To evaluate convergent validity of the test battery, the associations between the latent hot and cool EF factors and parent and teacher reports of children's inhibitory control and attention were studied. The model validating the EF assessment against report-based inhibitory control had acceptable fit [χ^2^ (127, *N* = 2827) = 348.02, *p* < 0.001, CFI = 0.946, RMSEA = 0.025 (0.022–0.028)]. Both hot and cool EF were significantly and positively related to report-based inhibitory control (see Figure 1A). The association between hot EF and report-based inhibitory control was not significantly different from the association between cool EF and report-based inhibitory control [ω (1) = 0.29, *p* = 0.588]. The model validating the EF assessment against report-based attention fitted the data well [χ^2^ (110, *N* = 2827) = 195.26, *p* < 0.001, CFI = 0.978, RMSEA = 0.017 (0.013–0.020)]. Both hot and cool EF latent factors were significantly positively related to report-based attention (see Figure 1B). However, the association with report-based attention was larger for cool compared to hot EF [ω (1) = 11.02, *p* < 0.001]. When only children in the center sample were included in the analyses, both models fitted the data well [report-based inhibitory control model: χ^2^ (116, *N* = 1802) = 219.00, *p* < 0.001, CFI = 0.949, RMSEA = 0.022 (0.018–0.027); report-based attention model: χ^2^ (100, *N* = 1802) = 159.73, *p* < 0.001, CFI = 0.973, RMSEA = 0.018 (0.013–0.023)]. The same pattern of results was found [report-based inhibitory control and hot EF: β = 0.33, *p* = 0.003; cool EF: β = 0.28, *p* = 0.034; ω (1) = 0.11, *p* = 0.741; report-based attention and hot EF: β = 0.22, *p* = 0.016; cool EF: β = 0.59, *p* < 0.001; ω (1) = 7.00, *p* = 0.008].

### Predictive validity

**Figure 2:**
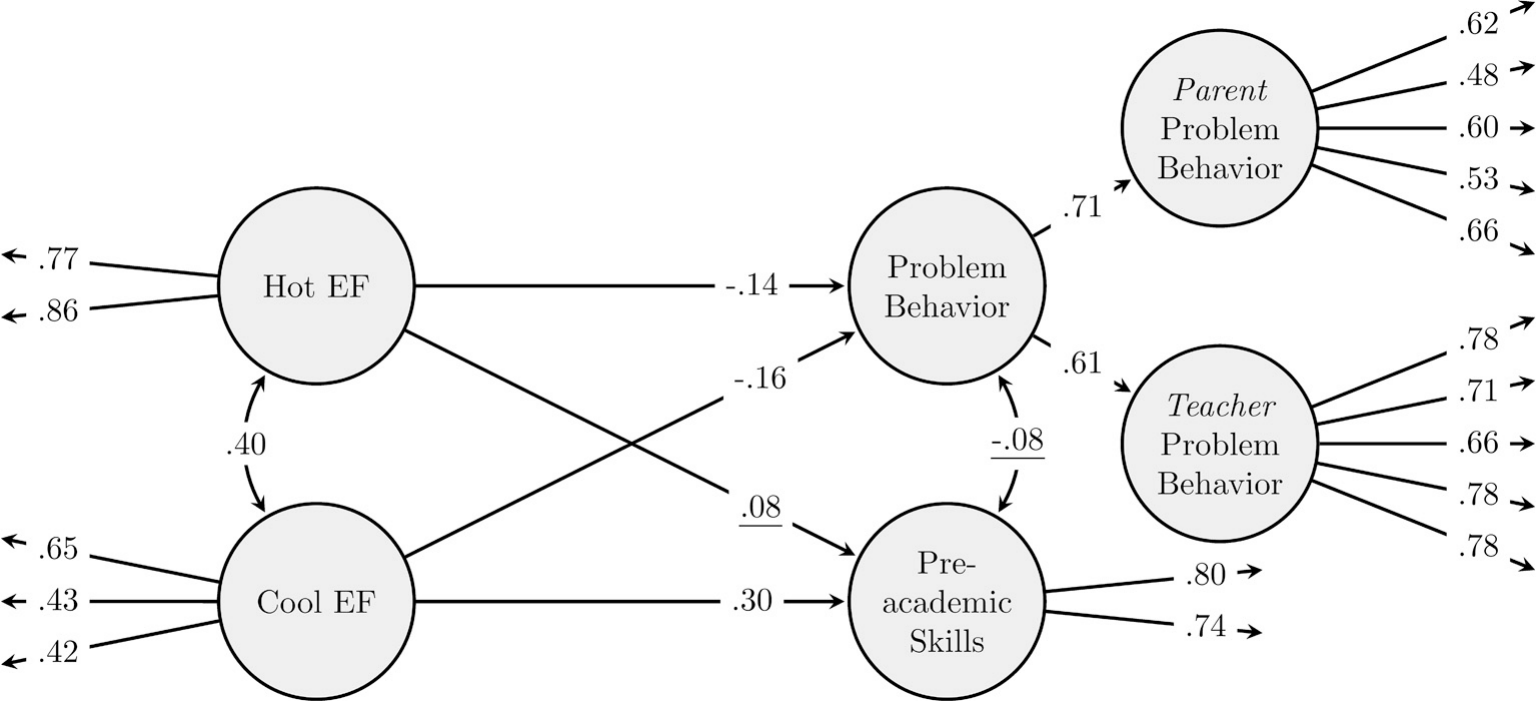
**Predictive validity model**. Age, gender, SES, home language, and cohort were included as covariates in the model. Values which are underlined are not significant.

In a final set of analyses, we investigated the predictive validity of the hot and cool EF constructs at age 2 years for behavioral functioning and pre-academic skills at age 3 years. Parent and teacher ratings on the BITSEA externalizing behavioral problem scale items were used as indicators to a latent multi-informant preschool externalizing behavior problem factor. Children's vocabulary and emerging math skills test scores were used to create a latent preschool pre-academic score. The predictive validity model had acceptable fit [χ^2^ (193, *N* = 2827) = 555.37, *p* < 0.001, CFI = 0.950, RMSEA = 0.026 (0.023–0.028)], as shown in Figure 2. Wave one cool EF was a significant predictor of both preschool externalizing behavior problems and emergent math and vocabulary as indicators of children's pre-academic skills at wave two. In contrast, wave one hot EF was a significant predictor of externalizing behavior problems, but not pre-academic skills, at wave two. The observed effects were unique effects: the effects of cool EF held while controlling for hot EF, and vice versa. When only children in the center cohort were included, model fit was acceptable χ^2^ (180, *N* = 1798) = 389.31, *p* < 0.001, CFI = 0.954, RMSEA = 0.025 (0.022–0.029). Results were similar to those of the full sample; the only difference was that the effect of hot EF on externalizing behavior problems was now no longer significant [problem behavior on hot EF: β = −0.15, *p* = 0.081; cool EF: β = −0.19, *p* = 0.038; pre-academic skills on hot EF: β = 0.01, *p* = 0.857; cool EF: β = 0.32, *p* < 0.001).

## Discussion

Executive function is an important predictor of academic achievement (Blair and Diamond, 2008), socio-emotional development (Carlson et al., 2004), and behavioral adjustment (Eisenberg et al., 2009; Espy et al., 2011) in the preschool period and beyond. The lack of psychometrically well-validated EF assessment instruments for very young children is a major obstacle to further progress our understanding of EF development in the early years (Blair and Ursache, 2010). The present study aimed to fill this void by investigating the psychometric quality of an executive function test battery for two-year-olds using confirmatory factor analysis. The EF task battery used in this study included measures of both cool EF (working memory, attention) and hot EF (delay of gratification). The battery comprised both new measures which were developed for the purposes of this study, and existing measures which were adapted for use in a large-scale field study. CFA analyses showed that (1) a two-factor hot and cool EF model fitted the data better than a one-factor EF model, (2) measurement invariance was supported across different subgroups of age, gender, home language, SES, and test setting, and (3) the test battery showed satisfactory criterion, convergent, and predictive validity.

Our first finding that a two-factor hot and cool EF model fitted the data better than a one-factor model is in line with results of most previous studies on preschoolers (Brock et al., 2009; Willoughby et al., 2011; Bassett et al., 2012; Kim et al., 2013). To the best of our knowledge, the current study is the first to provide support for a distinction between hot and cool EF in children as young as age 2 years through using CFA. However, it should be noted that the two indicators of the hot EF latent factor, the gift and snack delay task, were very similar in instruction and format. As such, these tasks may have loaded strongly on the same latent factor due to other factors than children's executive processing of affective information only. Future research could explore if similar results are obtained if more differentiated measures of hot EF are used with very young children.

Additional evidence for the differentiation between hot and cool EF factors at this young age comes from our predictive validity analyses. These analyses showed that the latent cool and hot EF factors were differentially predictive of children's outcomes 1 year later. In particular, the cool EF latent factor predicted both emergent math and vocabulary and externalizing behavior problems at age 3 years, whereas the hot EF factor only predicted externalizing behavior problems. These results support previous research showing similar relationships (Willoughby et al., 2011; Bassett et al., 2012; Kim et al., 2013; but see Brock et al., 2009). Note however, that our results were somewhat mixed: the association between hot EF and externalizing behavior problems was only observed in the full sample, and not in the center sample alone. In both analyses, the effect was relatively weak in strength. This could perhaps be explained by the fact that the two delay of gratification measures, which were used as indicators to the hot EF constructs, were not optimally distributed. In particular, in both tasks, about half of the children obtained the highest score. As such, there was not much differentiation between children at the higher end of the ability spectrum for hot EF, which could perhaps explain the relatively weak association with outcome measures.

Besides a CFA analysis comparing a one- and two-factor model, we tested measurement invariance of the latter, preferred, model across a number of different subgroups: younger vs. older toddlers, boys vs. girls, monolingual Dutch vs. other language groups, low/middle vs. high SES, and home-based assessment vs. center-based assessment settings. Strong measurement invariance was found, as the factor structure, factor loadings, intercepts of indicators, and associations between the hot and cool latent factors could all be constrained to equality across groups. This indicates that the tasks in our battery tap underlying EF ability in the same way in different subgroups of children, thus allowing for a fair comparison of latent hot and cool EF ability across these subgroups.

A further set of analyses showed significant relations between cool and hot EF, on the one hand, and gender, SES, age, home language, and test setting, on the other, supporting the criterion validity of the test battery. With respect to gender, girls outperformed boys on both EF constructs. Cross-cultural comparative studies have shown that child gender differences in EF, using the Head-Toes-Knees-Shoulders task (Ponitz et al., 2009) occur in some countries (i.e., the United States and Iceland), but not others (i.e., Taiwan, China, South Korea, Germany, and France) (Wanless et al., 2013; Gestsdottir et al., 2014). In the Netherlands, Huizinga and Smidts (2011) found higher EF scores for girls compared to boys in Dutch school-aged children and adolescents, using parent reports of EF. The results of the present study add to these findings by showing that such gender differences are already present well before school age, using a different assessment method, i.e., direct child assessments. Furthermore, we observed an effect of test setting on hot, but not cool EF. Children assessed at home achieved lower hot EF scores than children tested at their daycare center, after controlling for age, gender, SES, and home language. It is unclear, however, which factors could explain this effect. We also observed that children from non-monolingual Dutch homes scored lower on both cool and hot EF than their monolingual Dutch peers. The main subgroup in the non-monolingual Dutch sample consisted of non-Western immigrants, but results from the current study in this domain should be interpreted with caution, as this sample was very mixed. Our findings may indicate that differences in child rearing practices in different cultural groups can impact on EF development already at this young age. A recent cross cultural comparison across Syrian and German five- to twelve-year-old children showed that Syrian children performed less well on measures of sustained attention, visuospatial orienting, and executive function than their German peers (Sobeh and Spijkers, 2013). Alternatively, our findings could be due to differences in linguistic abilities across groups. Future studies are needed to investigate how cultural differences and associated child rearing practices, as well as linguistic differences, impact on EF development.

Apart from effects of age, gender and home language, an effect of SES was found such that children from low/middle SES backgrounds scored lower on cool EF than their high SES peers. The negative impact of low SES on EF in older children is well established (Noble et al., 2005, 2007). The present results add to these findings and show an effect of SES at a younger age, corroborating the findings by Hughes and Ensor (2005) that SES is related to EF at toddler age already. As previous studies have shown that a gap in academic achievement between children from low and high SES families persists over time (Heckman, 2006; Hackman et al., 2010), it seems especially important to design interventions to promote EF development in low SES children at a very young age. To date, preschool and parenting programs have most often focused on promoting EF in preschoolers, i.e., three- to five-year-olds, from disadvantaged families (e.g., Diamond et al., 2007; Neville et al., 2013). However, a recent review showed that attentional control and working memory training leads to more widespread transfer effects when given to younger children, potentially due to the fact that neural plasticity is larger in younger children (Wass et al., 2012). As such, there is a need to develop effective interventions to promote EF development in disadvantaged children even before preschool age and to design curricula for center-based education and care for young children that foster EF development.

In contrast to our findings regarding the influence of SES on cool EF, no SES effect was apparent on hot EF as measured with the snack and gift delay tasks. Previous studies have reported conflicting results regarding the role of SES in performance on delay tasks. For example, Li-Grining (2007) showed that there was no effect of socio-demographic risk on preschoolers' delay of gratification. In contrast, Evans and English (2002) found that eight- to ten-year-olds from low-income families performed less well on a delay of gratification measure than their peers from middle-income families. Thus, more research is necessary to investigate the role of SES on the development of delay of gratification, and whether effects of SES are specific to certain types of delay tasks or age ranges.

Finally, we found moderate correlations between the hot and cool EF factors and parent and teacher reports of children's attention and inhibitory control (Rothbart et al., 2003), supporting the EF tasks' convergent validity. Divergent validity was supported by the stronger association between reports of attention and children's cool EF compared to hot EF ability. Parent and teacher reports of children's attention mostly included items which covered the ability to remain focused and concentrate for longer periods of time. It is clear that such attentional focusing behavior was an important prerequisite for performing well in the working memory and selective attention measures. However, previous studies have shown that attention deployment is also an important factor in delay of gratification, or hot EF. Although we found evidence for this relation too, the association between hot EF and reported attention was weaker than between cool EF and reported attention. Potentially, in addition to the ability to remain focused, an alternative mechanism of selective attentional deactivation or distraction is more important for hot EF. For example, in the classic “marshmallow test,” children who distract themselves effectively from the single marshmallow which is put in front of them, are more effective at delaying gratification and waiting for a larger reward (i.e., two marshmallows at a later time), than children who focus on the single marshmallow instead (Mischel and Ebbesen, 1970; Peake et al., 2002). Future research is needed to investigate the association between these two types of attention deployment in different situations. The latent cool and hot EF factors were equally strongly related to reports of inhibitory control. This finding is not surprising, given that all three cool EF measures required some form of inhibitory control as well. In the selective attention task, children had to suppress pointing to distracting animals. Also, the six boxes visuospatial working memory task (Diamond et al., 1997) and memory for location task required children to search for hidden toys in identical boxes, and not re-open the boxes they had just opened. We observed that some toddlers sometimes made perseveration errors on these tasks, suggesting that inhibitory control processes play a role in performance, as has previously been observed in other search tasks for young children, such as the A-not-B task (Diamond et al., 1994).

The present study contributes to the extant literature about EF in early childhood in a number of ways. It is, to the best of our knowledge, one of the few validation studies to date that focused on children as young as 2 years, supporting the validity of an EF assessment already at this young age. Moreover, it used CFA to investigate whether the current EF assessments represented a one- or two-factor structure, and thoroughly investigated measurement invariance across various subgroups. Importantly, unlike previous studies, our study was conducted in a large, nation-wide sample, involving over 2000 children and including a large number of children from low/middle SES families and families in which other languages than Dutch were spoken, increasing the external validity of the results. Furthermore, children's EF task measures were triangulated by independent parent and teacher reports on children's behavior in naturalistic settings at home and in daycare, revealing considerable shared variance, supporting the validity of the EF measures. Moreover, we assessed predictive validity of the test battery, showing significant associations between children's EF at age 2 years and behavioral problems and pre-academic skills (i.e., vocabulary and emergent math) at age 3 years.

There are, however, also a number of limitations. First of all, missing data were substantial, especially regarding SES and parent and teacher reports of children's behavior. Second, it would have been beneficial to include more tasks in each domain. In the cool EF domain, inclusion of measures of shifting and inhibitory control would have allowed for a more comprehensive construct. However, in our experience, selecting tasks to assess shifting and inhibitory control for such young children is challenging. These tasks often rely on “if-then” rules (e.g., Go-NoGo tasks in which the child is instructed to press a key if stimulus X is shown and withhold their response when stimulus Y is shown) and such rules are often too challenging for two-year-olds (Zelazo and Reznick, 1991), although shifting measures have been successfully administered to two-year-olds in some studies (e.g., Hughes and Ensor, 2007; Beck et al., 2011). In the hot EF domain, more delay tasks with a different administration format would have provided a more pure hot EF latent construct. However, decisions regarding measurement selection were made with testing time in mind; for the purposes of this large-scale field study with very young children, test time was limited. Finally, we used non-standard versions of the ECBQ and BITSEA questionnaires for validation purposes.

A number of implications arise from the current study. First, our results showed that the assessment of EF through using multiple measures and modeling latent constructs showed satisfactory to good psychometric properties in this very young sample. Thus, the current study shows that EF can be assessed reliably in children as young as 2 years of age. As in the study by Willoughby et al. (2010) with three-year-olds, we observed that the associations among separate EF tasks were relatively weak. However, all tasks loaded significantly onto their latent factors, and latent factors were in turn significantly related to a number of outcome measures, with substantial effect sizes. Our findings thus support Willoughby et al.'s (2010) conclusion that, especially for very young children, it is recommended to use multiple EF measures to be able to construct latent factors. This way, the influence of measurement error is reduced and the reliability of the EF assessment is increased (Willoughby and Blair, 2011). Second, the current study shows that, even at the age of 2 years, EF can be meaningfully differentiated in a cool and hot component. Thus, for applied research in which an assessment of EF is included to predict children's outcomes across multiple domains, inclusion of both hot and cool EF measures is recommended.

To conclude, our EF task battery for two-year-old children in the Netherlands showed satisfactory psychometric quality and criterion, convergent and predictive validity. We are currently investigating data from the children in this sample at age three, four, and five years, to investigate their development of EF from the toddler through to the preschool years. The current instruments offer new opportunities for investigating EF development in early childhood and for evaluating interventions targeted at improving EF from a young age.

## Author contributions

Hanna Mulder developed, piloted, and implemented the test battery; wrote introduction, sample, and task descriptions, and discussion; reviewed and revised analyses and results section. Huub Hoofs provided an initial draft of the manuscript as his MSc thesis; wrote analyses and results section. Josje Verhagen co-developed, piloted and implemented the test battery; reviewed and revised the manuscript. Ineke van der Veen project design; general project methodology; questionnaire design. Paul P. M. Leseman principal investigator of the project; reviewed and revised the manuscript.

### Conflict of interest statement

The authors declare that the research was conducted in the absence of any commercial or financial relationships that could be construed as a potential conflict of interest.

## Footnotes

^1^The research reported in this article involves healthy human participants, and does not utilize any invasive techniques, substance administration or psychological manipulations. Therefore, compliant with Dutch law, this study only required, and received approval from our internal faculty board (Faculty's Advisory Committee under the Medical Research (Human Subjects) Act (WMO Advisory Committee) at Utrecht University.
